# Taxonomy and distribution of some katydids (Orthoptera Tettigoniidae) from tropical Africa

**DOI:** 10.3897/zookeys.524.5990

**Published:** 2015-09-30

**Authors:** Bruno Massa

**Affiliations:** 1 Department of Agricultural and Forest Sciences, University of Palermo, Viale Scienze 13, 90128 Palermo, Italy.

**Keywords:** Distribution, taxonomy, tropical Africa, new species, synonymies

## Abstract

Results of the study of specimens collected in tropical Africa and preserved in different European collections and museums are reported and extensively illustrated. The following three new species are described: *Horatosphaga aethiopica* **sp. n.**, *Dapanera occulta* **sp. n.** and *Cestromoecha laeglae* **sp. n.** In addition, new diagnostic characters or distributional data for *Ruspolia differens* (Serville, 1838), *Thyridorhoptrum senegalense* Krauss, 1877, *Horatosphaga leggei* (Kirby, 1909), *Horatosphaga linearis* (Rehn, 1910), *Preussia lobatipes* Karsch, 1890 and *Dapanera eidmanni* Ebner, 1943 are reported. Finally, *Symmetropleura plana* (Walker, 1869) is proposed to be transferred to the genus *Symmetrokarschia* Massa, 2015, *Conocephalus carbonarius* (Redtenbacher, 1891) to the genus *Thyridorhoptrum* Rehn & Hebard, 1915; the genus *Gonatoxia* Karsch, 1889 is proposed to be synonymized with *Dapanera* Karsch, 1889.

## Introduction

The present paper is the result of the study carried out on material collected in tropical Africa by different collectors and preserved in various museums; it follows other three papers on the same subject (Massa 2013, 2014, 2015). Tropical Africa is a very rich area of Orthoptera, order of insects that in these regions have reached a very high degree of morphological diversity. Concerning katydids, many studies have been carried out since 1800 in this wide geographic area, but this group of Ensifera still hides many unknown taxa. Here taxonomy and distribution of some selected species are discussed and new taxa are described.

## Material and methods

Series of tropical African specimens kindly obtained from Philippe Moretto were studied and identified; further specimens were examined in the below cited museums or loaned from them.

### Abbreviations used in this paper

BMCP Bruno Massa Collection, University of Palermo;

ISAM Iziko South African Museum, Cape Town;

MfN Museum für Naturkunde, Berlin;

MNCN Museo Nacional de Ciencias Naturales, Madrid;

MRT Museo Regionale di Storia Naturale, Terrasini (Palermo);

MSNG Museo Civico di Storia Naturale ‘G.Doria’, Genoa;

MZR Museo di Zoologia Università La Sapienza, Rome.

Some specimens were photographed with a Nikon Coolpix 4500 digital camera, mounted on a Wild M5 Stereomicroscope or Leika MZ75, and photos were integrated using the freeware CombineZP (Hadley 2008). Mounted specimens were measured with a digital calliper (precision 0.01 mm); the following measures were taken (all measurements in mm): Body length: dorsal length from the head to the apex of the abdomen, ovipositor excluded in females; Pronotum length: length of the pronotum along dorsal median line; Pronotum height: maximum height of the pronotum; Hind femur: length of hind femur; Tegmina: length of tegmina; Ovipositor: maximum length.

## Results

### Fam. Tettigoniidae Krauss, 1902

#### Subfam. Conocephalinae Burmeister, 1838

##### Tribe Copiphorini Karny, 1912

###### Ruspolia differens

Taxon classificationAnimaliaOrthopteraTettigoniidae

(Serville, 1838)

####### Material examined.

Seychelles, Silhouette Is. 27.VI-3.VII.1988, F.A. Repetti (1♂) (MSNG).

####### Distribution.

Angola, Ghana, Ivory Coast, Central African Republic, Zaire, Rwanda, Kenya, Uganda, Tanzania, Rhodesia, Zanzibar, Mauritius, Madagascar (Bailey 1975, Bailey and McCrae 1978).

####### Remarks.

*Ruspolia differens* is a very widespread species throughout tropical Africa, including also some islands of the Indian Ocean. Its presence in the Seychelles archipelago is possibly explained as a passive importation.

##### Tribe Conocephalini Burmeister, 1838

###### Genus *Thyridorhoptrum* Rehn & Hebard, 1915

Rehn and Hebard (1915) described the genus *Thyridorhoptrum* with these characters: pronotum more abbreviate than in the American genus *Orchelimum* Krauss, 1877, very narrow lateral lobes of pronotum, an extremely large male stridulatory field of tegmina, with a large speculum (at least two-thirds that of the whole stridulatory field; the Latin name *Thyridorhoptrum* means window tambourine), bidentate male cerci, and broad fluting of the lateral surfaces of the ovipositor, abruptly terminating shortly proximad of the apex. They included only the species *Thyridorhoptrum senegalense* (Krauss, 1877). Later, Pitkin (1977), on the basis of much material coming from West, central, East and South regions of Africa, revised the genus, and described another species, *Thyridorhoptrum baileyi*, highlighting that both species of the genus may have two different forms, one with large mirror and another with small mirror, that could belong to different taxa. Finally, a third undescribed species has been recorded by Naskrecki (1999) from Ghana. Among material collected by Philippe Moretto in the Ivory Coast there is a species matching with the description of *Xiphidium carbonarium* Redtenbacher (1891), currently listed as Conocephalus (Conocephalus) carbonarius by Eades et al. (2015); it is here proposed to ascribe it to the genus *Thyridorhoptrum*.

####### Thyridorhoptrum senegalense

Taxon classificationAnimaliaOrthopteraTettigoniidae

Krauss, 1877

######## Material examined.

Ivory Coast, Tuba, Biémasso 7-11.VII.2014 (UV), P. Moretto (1♀); Ivory Coast, Tuba, Biémasso forest 7-11.VII.2014, P. Moretto (1♀); Ivory Coast, Man, Mt. Tonkoui (1200 m) 24-27.XI.2014 (UV), P. Moretto (1♀); Burkina Faso, Borolo, Ft. Sorobouli 1-4.VII.2013 (UV), P. Moretto (1♀) (BMCP).

######## Remarks.

The specimens above listed have 1 spine on outer genicular lobe of fore femora and on inner and outer genicular lobes of hind femora.

####### Thyridorhoptrum carbonarium

Taxon classificationAnimaliaOrthopteraTettigoniidae

(Redtenbacher, 1891) comb. n.

Figs 1–7 , 8–12

######## Material examined.

Ivory Coast, Man, Mt. Tonkoui (1200 m) 7°26'58.46"N, 7°39'01.14"W 1–2.VII.2014 (UV), P. Moretto (1♂, 1♀); Ivory Coast, Man, Mt. Tonkoui (1200 m) 28.VI-1.VII.2014 (UV), P. Moretto (5♂, 4♀); Ivory Coast, Man, Mt. Tonkoui (1200 m) 24–27.XI.2014 (UV), P. Moretto (2♂, 3♀); Ivory Coast, Korhogo Village (347 m) 9°25'07.02"N, 5°36'59.41"W 13–15.VII.2014 (UV), P. Moretto (1♂) (BMCP).

######## Previous records.

Redtenbacher (1891) described this species from Accra (Ghana). Chopard (1954) reported it as *Conocephalus* from Guinea, Ragge (1967) listed it again as *Conocephalus* from Democratic Republic of Congo, and Naskrecki (2008, 2009) from Ghana.

######## Redescription.

**Figures 1–7.**
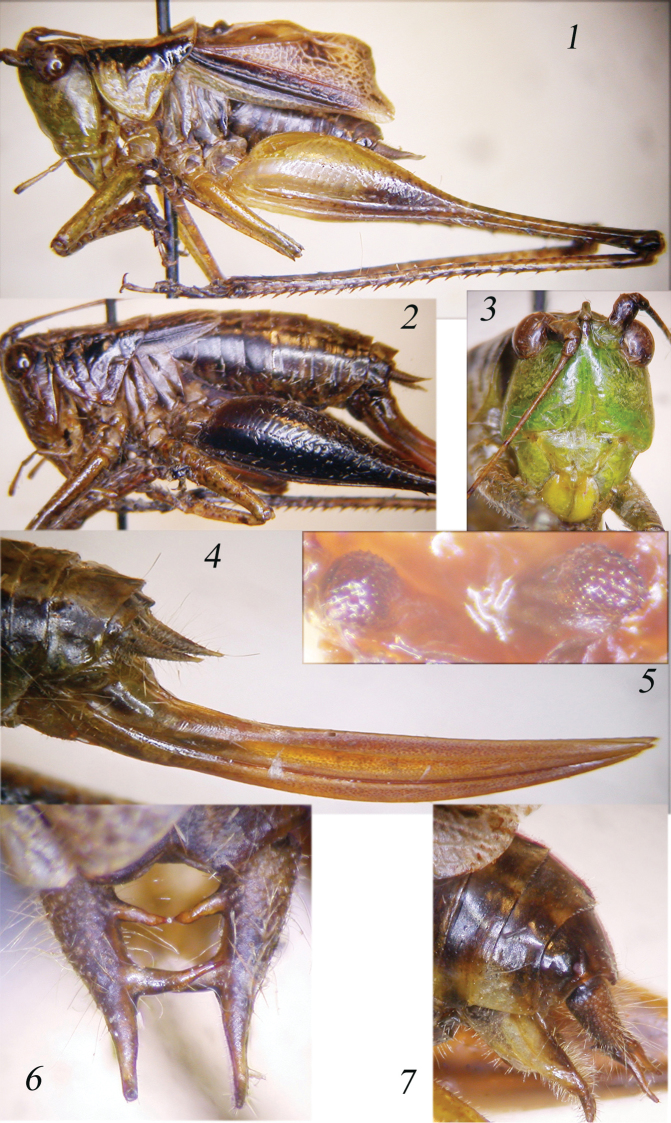
*Thyridorhoptrum carbonarium* comb. n. Lateral view of male (**1**) and of female (**2**); face of the male (**3**); ovipositor (**4**); titillators (**5**); dorsal (**6**) and lateral view of cerci (**7**).

**Figures 8–12.**
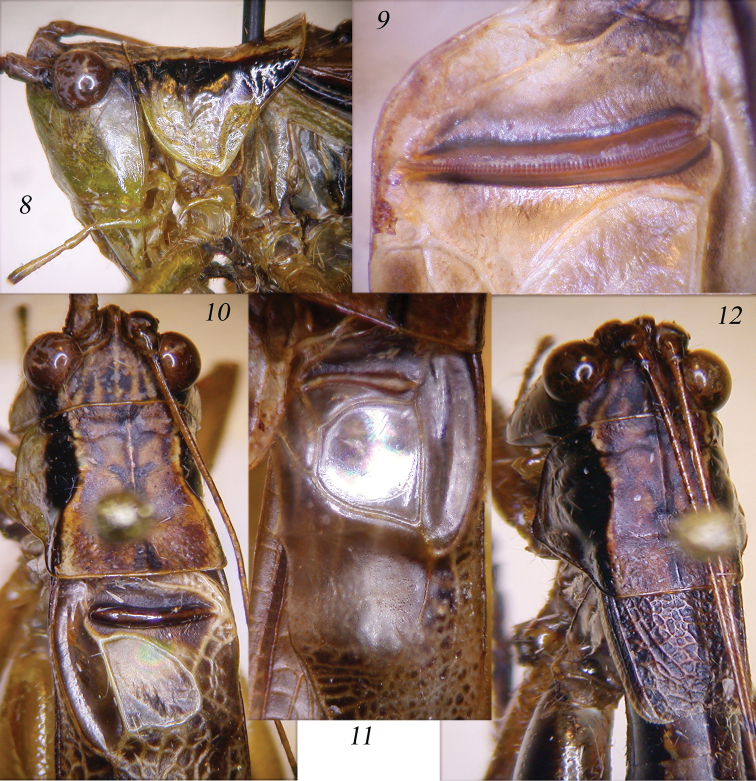
*Thyridorhoptrum carbonarium* comb. n. Lateral view of the head and pronotum of male (**8**); stridulatory file (**9**); dorsal view of head, pronotum and stridulatory area of left tegmen of male (**10**); mirror of right tegmen (**11**); dorsal view of head, pronotum and tegmina of female (**12**).

When Redtenbacher (1891) described this species, he highlighted that two spines are present on the prosternum. Thus, it cannot be ascribed to the subgenus *Conocephalus* Thunberg, 1815, that lacks these spines. However, characters of male and female are more matching those of the genus *Thyridorhoptrum* than those of *Anisoptera* Latreille, 1829, to which the species has been ascribed by Kirby (1906). The characters are: Fastigium of vertex very narrow and raised between antennae (Fig. 3). Antennae twice longer than body. Eyes round (Figs 8, 10, 12). Fore coxae armed with a long spine, fore and mid femora unarmed, hind femora with 3 spines on outer lower margin. 1 small spine on the outer genicular lobe of fore femora and on inner and outer hind femora. Fore and mid tibiae with 4-5 spines on lower margins + 1 spur on each side, dorsal margins unarmed. Tympanic auricles closed. Hind tibiae with 8-10 spines on both lower margins + 2 spurs on each side; upper margins with many spines + 1 spur on each side. Sternum armed with two spines. Tegmina as long as abdomen or little shorter, clearly inflated, hind wings nearly rudimentary, as long as tegmina but very narrow. The stridulatory area of the left tegmen is accentuated by a swelling of the rib, which gives an undulating appearance when viewed along the lateral plane of the wing (Figs 1, 10). Stridulatory file of left tegmen as in Fig. 9; row of teeth not much curved, differentiated into very dense teeth at the dorsal and proximal ends and large and spaced teeth in the central section. The mirror of right tegmen is slightly rounded (Fig. 11). Cerci finely pointed, with two inner spines, the first shorter than the second, both are smooth (Fig. 6). Sub-genital plate concave with small styli (Fig. 7). Titillators, previously undescribed, are small and round, with a very wrinkled surface (Fig. 5). Female pronotum not raised, with metazona matt and roughly punctate (Fig. 12), micropterous, with very short tegmina, narrow hind wings, just exceeding tegmina (Figs 2, 12). Ovipositor gently up-curved (Fig. 4), sub-genital plate triangular with straight margin. Cerci conical.

######## Colour.

Brown with green parts, with a clear darkish stripe on head and pronotum, continuing on fore wings. Frons green, hind legs with darkish markings in the distal parts of femora and basal parts of tibiae, in some specimens darkish outer face of hind femora (Figs 1, 2).

######## Measurements.

See Table 1. *Thyridorhoptrum carbonarium* is a small species with very short wings (mainly in the females) and short ovipositor.

**Table 1.** Measurements of *Thyridorhoptrum carbonarium* comb. n. compared with the two forms (large- and small-mirror) of *Thyridorhoptrum senegalense* and *Thyridorhoptrum baileyi* (after Pitkin 1977). For all species the min-max range is reported, for *Thyridorhoptrum carbonarium* in parenthesis the mean value is also reported.

Species	Total length	Pronotum length	Length of hind femora	Length of tegmina	Length of ovipositor
*Thyridorhoptrum senegalense* large mirror	27.6–40.0 (♂) 34.0–45.9 (♀)	4.2–5.8 (♂) 4.0–5.6 (♀)	14.5–19.5 (♂) 15.6–22.3 (♀)	21.0–31.0 (♂) 25.1–35.9 (♀)	9.9–12.1
*Thyridorhoptrum senegalense* small mirror	28.0–31.9 (♂) 34.1–41.8 (♀)	4.7–5.5 (♂) 4.6–5.7 (♀)	15.8–17.9 (♂) 18.1–22.1 (♀)	19.4–25.2 (♂) 25.6–33.0 (♀)	11.2–14.1
*Thyridorhoptrum baileyi* large mirror	29.2–43.2 (♂) 33.6–41.9 (♀)	4.1–6.0 (♂) 4.0–5.0 (♀)	13.7–19.4 (♂) 16.1–19.9 (♀)	24.1–34.6 (♂) 26.6–33.6 (♀)	8.8–10.7
*Thyridorhoptrum baileyi* small mirror	20.4–34.9 (♂) 30.5–37.3 (♀)	4.2–5.4 (♂) 4.8–5.6 (♀)	13.6–19.6 (♂) 18.1–21.6 (♀)	14.1–27.0 (♂) 21.1–27.4 (♀)	12.0–13.9
*Thyridorhoptrum carbonarium* comb. n.	13.5–15.0 (14.0) (♂) 16.0–18.5 (17.3) (♀)	3.7–4.4 (4.1) (♂) 4.1–4.5 (4.3) (♀)	14.0–15.8 (15.2) (♂) 16.2–18.2 (17.5) (♀)	7.4–8.5 (8.0) (♂) 3.2–3.6 (3.4) (♀)	8.7–9.7 (9.2)

######## Diagnosis.

No brachypterous species of *Thyridorhoptrum* are known. Both *Thyridorhoptrum senegalense* and *Thyridorhoptrum baileyi* are long-winged. The mirror of male right tegmen of *Thyridorhoptrum carbonarium* is similar to that of the small mirror form of *Thyridorhoptrum senegalense*, while in *Thyridorhoptrum baileyi* is more triangular. The stridulatory files are differently shaped in the other two species (see Pitkin 1977). The first inner spine of cerci in *Thyridorhoptrum senegalense* and *Thyridorhoptrum baileyi* has a serrated apex, while it is smooth in *Thyridorhoptrum carbonarium*. The ovipositor of *Thyridorhoptrum carbonarium* is gently up-curved, while in the other two species it is quite strongly up-curved.

######## Habitat.

According to Chopard (1954), this species was collected in Guinea in forest habitats. Mt. Tonkoui is a forested mountain of Ivory Coast, with an average elevation of ca. 1,000 m a.s.l. It is covered by a tropical Moist Forest with evergreen broadleaved species. Nevertheless, *Thyridorhoptrum carbonarium* is not strictly linked to forest habitats, because it has also been collected at light in the village of Korhogo (347 m). It seems that the main geographical feature is an average annual rainfall of ca. 1,200 mm, with the rainiest months being May to October, when adults of *Thyridorhoptrum carbonarium* are active.

#### Subfam. Phaneropterinae Burmeister, 1838^1^

##### Tribe Acrometopini Brunner von Wattenwyl, 1878

###### Genus *Horatosphaga* Schaum, 1853

According to Ragge (1960) the genus *Horatosphaga* is characterized as follows: basal part of MA of fore wings developed into longitudinal concavity with reduced venation, Cu_1a_ area basally enlarged, cross veins (mainly in costal and anterior medial areas) arranged in closely parallel fashion, forming web-like pattern, R_s_ or its branches ending at tip of wings. Fastigium of frons almost reaches to top of antennal scrobes, tympanic auricles of fore tibiae often inflated. Females differ by fastigium of vertex sloping steeply to frons, pronotum sometimes with lateral carinae, tympana of fore tibiae not inflated, fore wings unmodified and hind wings rudimentary.

Twenty-nine species are currently listed within the genus *Horatosphaga*, of which three have been described by Hemp (2002, 2006, 2007), after the revision of Ragge (1960). In addition, Hemp et al. (2010) have described the genus *Altihoratosphaga*, including four species, three previously included in the genus *Horatosphaga*, *Altihoratosphaga montivaga* (Sjöstedt, 1910), *Altihoratosphaga nomima* (Karsch, 1896) and *Altihoratosphaga nou* (Hemp, 2006), and another, *Altihoratosphaga anangensis* Hemp, 2010, newly described in Hemp et al. (2010).

Characters of *Altihoratosphaga* are round tegmina with reduced venation and scattered black spots (except for *Altihoratosphaga nomima*), vestigial alae, shape of pronotum verrucose in most species, and emarginate tenth abdominal tergite (only found similarly in the fully winged *Horatosphaga concava* Ragge, 1960). Females may be recognized by their slender, long, and slightly upcurved ovipositor. Both sexes in species of *Altihoratosphaga* are rather plump and dark green in colour, with rounded broad wings lacking web-like venation, whereas typical *Horatosphaga* are more slender, especially the males, and are mostly light green in colour, with more elongated wings, and with web-like venation in the male forewings. *Horatosphaga* is a very heterogeneous genus, with variable characters among species (e.g.: length of wings, sexual dimorphism, ovipositor shape, etc.), and following Ragge (1960), several species within this genus remain to be described. Here a new Ethiopian species is described, showing the characters of the genus.

####### Horatosphaga leggei

Taxon classificationAnimaliaOrthopteraTettigoniidae

(Kirby, 1909)

Figs 13 , 15 , 25

######## Material examined.

Democratic Republic of Congo, Goma 3.I.1967, T. De Stefani (1♂); same data 11–12.XII.1967 (2♂); same data 21.XII.1967 (2♂); same data 25.XII.1967 (1♂); same data 29.XII.1967 (1♂); same data 31.XII.1967 (1♂); Democratic Republic of Congo, Bukavu 7.VIII.1967, T. De Stefani (1♂); same data 6.III.1969 (1♂); Democratic Republic of Congo, Mt. Kanzi (2000 m) 22.III.1970, T. De Stefani (1♂) (MRT).

######## Remarks.

**Figures 13–16.**
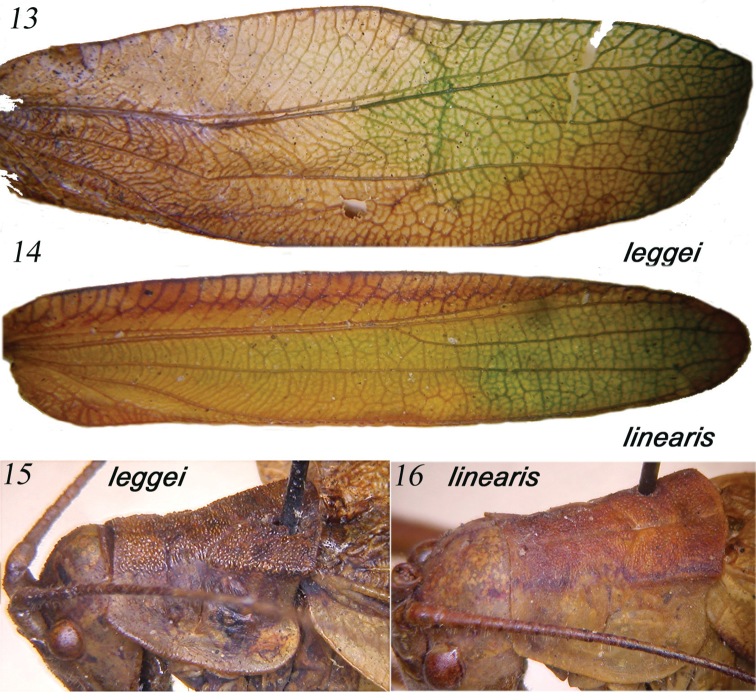
Right tegmen of females of *Horatosphaga leggei* (**13**) and *Horatosphaga linearis* (**14**); lateral view of female pronotum of *Horatosphaga leggei* (**15**) and *Horatosphaga linearis* (**16**).

The right tegmina of specimens listed (both males and females) (Fig. 13) have venation as depicted by Ragge (1960), who also wrote that the female has the pronotum with well-developed lateral carinae in metazona. However, the female pronotum also has a verrucose surface of disc, not cited by Ragge (1960) (Fig. 15). *Horatosphaga leggei* has a stridulatory file composed of 92-95 regularly placed teeth (Fig. 25).

######## Distribution.

According to Ragge (1960) *Horatosphaga leggei* is the most common species of the genus from East Africa to the Democratic Republic of Congo.

####### Horatosphaga linearis

Taxon classificationAnimaliaOrthopteraTettigoniidae

(Rehn, 1910)

Figs 14 , 16

######## Material examined.

Democratic Republic of Congo, Bukavu 28.III.1969, T. De Stefani (1♀); 10 Km N-NW Bukavu 3.VIII.1970, T. De Stefani (1♀) (MRT).

######## Remarks.

The female of this species is well characterized by the venation of the right tegmen (Fig. 14) and slender ovipositor (Ragge 1960); the female pronotum has a verrucose surface of disc (Fig. 16), previously not cited (cf. Ragge 1960), but less so than in *Horatosphaga leggei*.

####### Horatosphaga aethiopica sp. n.

Taxon classificationAnimaliaOrthopteraTettigoniidae

http://zoobank.org/C13E52EB-70F7-4D4F-AA03-BD41E1D416DA

Figs 17–21 , 22–24

######## Material examined and depository.

Ethiopia, Omo river, El Dire 5°06'21.45"N, 36°51'08.77"E (950 m) 21.V.1939 (Expedition E. Zavattari) (♂ holotype, ♀ allotype, ♂ paratype); Ethiopia, Omo river, El Dire (950 m) 19.V.1939 (Expedition E. Zavattari) (♀ paratype); Ethiopia, Omo river, Calam 4°41'20.08"N, 35°39'58.46"E (370 m) 14.VIII.1939 (Expedition E. Zavattari) (♀ paratype)) (MZR).

######## Colour.

**Figures 17–21.**
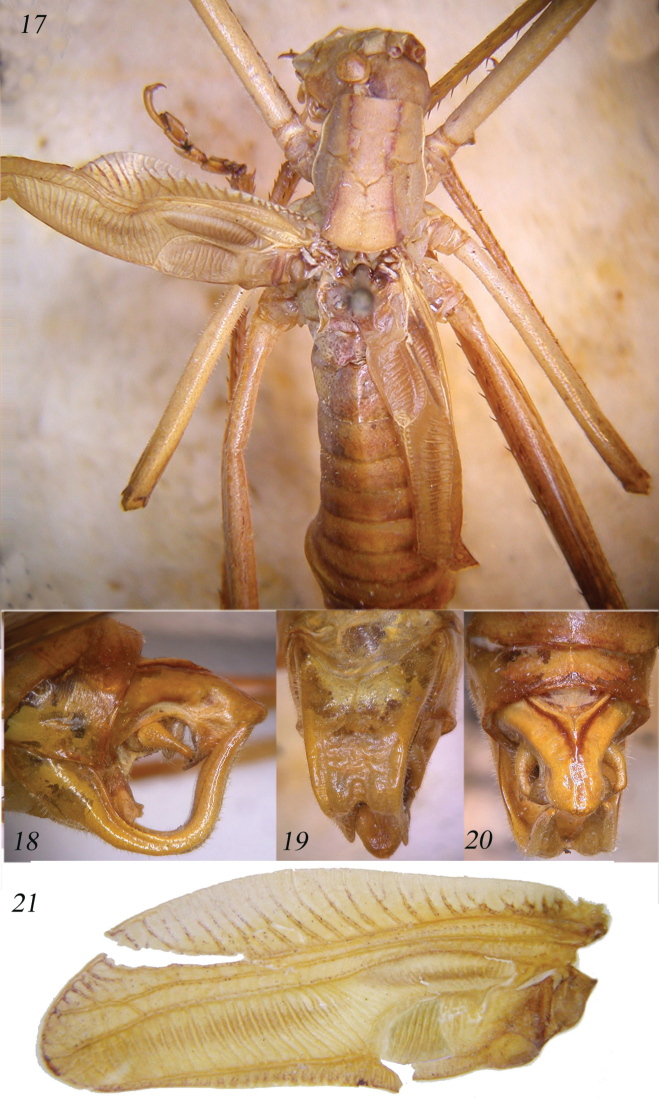
*Horatosphaga aethiopica* sp. n. Dorsal view of male (**17**); lateral view of last abdominal tergites of male (**18**); sub-genital plate of male (**19**); dorsal view of last abdominal tergite, cerci and appendices of the sub-genital plate of male (**20**); left tegmen of male (**21**).

**Figures 22–25.**
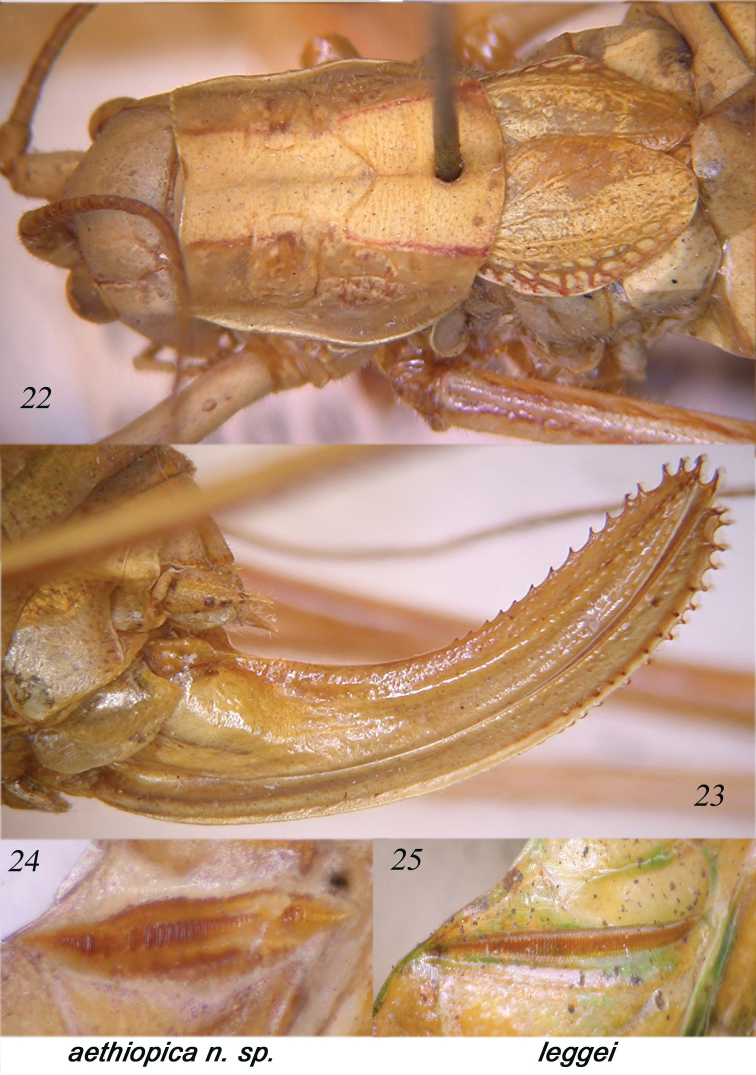
Dorsal view of head, pronotum and tegmina of female of *Horatosphaga aethiopica* sp. n. (**22**); lateral view of the ovipositor (**23**); stridulatory file of male of *Horatosphaga aethiopica* sp. n. (**24**); stridulatory file of *Horatosphaga leggei* (**25**).

Yellowish (alive specimens may show different colour) (Figs 17, 22).

######## Description.

Male. Head and antennae: fastigium of vertex very narrow, furrowed above, separated from the tuberculated fastigium of frons. Eyes rounded, well projecting. Legs long. Fore coxae unarmed. Fore tibiae furrowed on upper margin, distinctly widening above tympanum, which is closed on inner and on outer sides, tympanic auricles inflated. Fore femora unarmed, fore tibiae with 11 spines plus 1 spur on inner and outer ventral margins, 1 spur on inner and outer dorsal margins, mid femora unarmed, mid tibiae with 14 spines on inner and outer ventral margins, plus 1 spur on each side and 1 spur on both sides of dorsal margins, hind femora unarmed, hind tibiae with many spines on ventral and dorsal margins and 3 spurs on each side. Thorax: pronotum little narrowing anteriorly, little raised posteriorly, anterior margin straight, posterior margin rounded, humeral sinus absent, lobes of pronotum rounded and low. Tegmina shorter than abdomen, with pointed apices, their web-like venation very simple, cross-veins of area MA are arranged in a parallel fashion, a bit arcuate in inner part (Fig. 21). Hind wings rudimentary and linked to metanotum. Stridulatory region of left tegmen short and inflated, stridulatory file composed of 20-25 widely spaced teeth, of different size (Fig. 24). Abdomen: tenth tergite greatly enlarged and completely concealing supra-anal plate, in lateral view similar to a raptor beak (Figs 18–20); sub-genital plate long, up-curved and deeply divided into two lobes reaching margin of the tenth tergite and curved a bit backwards at end (Fig. 19); styli absent. Cerci short, in-curved, with a small apical spine (Fig. 20).

Female. As the male, but tegmina reduced to two small overlapping scales, not exceeding first abdominal tergite, but showing a residual web-like venation (Fig. 22). Cerci conical and pointed. Ovipositor up-curved and provided with many denticles on upper and lower margins (Fig. 23).

######## Measurements.

See Table 2. *Horatosphaga aethiopica* is characterized by its very small size, compared to related species, in particular in the length of tegmina and ovipositor.

**Table 2.** Measurements of *Horatosphaga aethiopica* sp. n. and the related three species *Horatosphaga ruspolii*, *Horatosphaga vicina* and *Horatosphaga diminuta* (after Ragge 1960). For all species the min-max range is reported.

Species	Total length	Pronotum length	Length of hind femora	Length of tegmina	Length of ovipositor
*Horatosphaga ruspolii*	37.7–44.7 (♂) 25.0–26.6 (♀)	4.7–5.7 (♂) 5.9 (♀)	24.7–28.4 (♂) 29.3 (♀)	29.4–35.2 (♂) 17.9–19.0 (♀)	9.8–10.3
*Horatosphaga vicina*	32.0–35.2 (♂) 23.5–27.0 (♀)	6.9–7.1 (♂) 7.2–7.4 (♀)	29.2–31.4 (♂) 25.4 (♀)	25.0–26.9 (♂) 14.8–17.0 (♀)	13.3
*Horatosphaga diminuta*	26.3–32.0 (♂) 20.5–26.1 (♀)	4.7–6.1 (♂) 5.6–6.6 (♀)	22.7–28.2 (♂) 23.4–27.8 (♀)	18.9–25.7 (♂) 13.3–17.8 (♀)	9.8–10.8
*Horatosphaga aethiopica*	20.8–22.1 (♂) 20.5–22.0 (♀)	4.7–4.8 (♂) 5.0–5.5 (♀)	19.5–20.0 (♂) 21.3–21.5 (♀)	11.6–12.0 (♂) 3.1–3.2 (♀)	8.2–9.1

######## Diagnosis.

Concerning the affinities between this and related species, there are only three *Horatosphaga* with the male having the tenth abdominal tergite greatly enlarged and completely concealing the supra-anal plate, namely *Horatosphaga ruspolii* (Shulthess, 1898), *Horatosphaga diminuta* (Chopard, 1954) and *Horatosphaga vicina* (Chopard, 1954), all described from Kenya. *Horatosphaga ruspolii* has fully developed wings, while the other two have reduced fore wings and rudimentary hind wings. According to Ragge (1960) *Horatosphaga ruspolii* lives also in the eastern Ethiopia, in the area between Ethiopia and Somalia, and in Uganda. Its external genitalia are very variable, but appendices of the sub-genital plate are simply up-curved and shorter than those of *Horatosphaga aethiopica* sp. n. However, Ragge (1960) suspected that *Horatosphaga diminuta* could be a brachypterous form of *Horatosphaga ruspolii*, and considered also that *Horatosphaga vicina* could be a large form of *Horatosphaga diminuta*, that also Chopard (in Chopard and Mc Kevan 1954) considered almost an exact repetition of *Horatosphaga vicina* on a rather smaller scale. *Horatosphaga aethiopica* sp. n., which is smaller than the above three species (see Ragge 1960 and Table 2), differs from the previous species not only by its external male genitalia, but also by the high reduction of wings, mainly in the female, a character that forces individuals to an important isolation. The reduction of wings probably was also the cause for a very reduced stridulatory file. Regarding the reduction of the stridulatory file related to the wing reduction, it is possible that also *Horatosphaga diminuta* has a different stridulatory file compared to the fully developed wings of *Horatosphaga ruspolii*, and this should result in a different song, an important specific barrier.

######## Etymology.

The Latin name *aethiopica* is a female adjective meaning “living in Ethiopia”.

######## Discussion.

In 1939, between March and September, Zavattari (1943) carried out an expedition to the territory of the Omo river in Ethiopia. During that trip, participants reached the northern part of the Turkana lake, where they collected also some Orthoptera. Among them there was the series of *Horatosphaga* specimens listed above. This new taxon is remarkably different from all related taxa. Indeed, in none of the species known till now, the tegmina of the female are so much reduced to two small scales, as in *Horatosphaga aethiopica* sp. n. Concerning the tribe Acrometopini, the characters of this *Horatosphaga* species and those of other related species as provided by Hemp (2011) and Hemp et al. (2010) modify and update the key in Ragge (1960) partially based on the ratio between the length of the pronotum to that of the tegmina.

##### Tribe Phaneropterini Burmeister, 1838

###### Symmetrokarschia plana

Taxon classificationAnimaliaOrthopteraTettigoniidae

(Walker, 1869) comb. n.

Figs 26–30

####### Material examined.

South Africa, Kwa Zulu-Natal, Nhandla Forest, I.1937 (2♂) (ISAM).

####### Remarks.

Walker (1869) described *Phaneroptera plana* from Kwa Zulu-Natal (South Africa). Later, Kirby (1906) transferred the taxon to the genus *Tylopsis* Fieber, and Ragge (1964) placed it in the genus *Symmetropleura* Brunner, 1878. Another species described by Chopard (1955), *Catoptropteryx latipennis* from Cape Province, Tsitzikama forest, was synonymized by Huxley (1970) with *Symmetropleura plana*. The genus *Symmetropleura* was based on a Neotropical type-species, *Symmetropleura laevicauda* Brunner, 1878 and contained three further Neotropical and three African species. However, Ragge (1968, 1980) pointed out that *Symmetropleura* is a New World genus, occurring in South America, Mexico and the Eastern USA, and that the African species are neither very similar to each other nor to the Neotropical type-species of the genus. Finally, Massa (2015) described two new genera for two African species: *Symmetrokarschia africana* (Brunner von Wattenwyl, 1878) and *Symmetroraggea dirempta* (Karsch, 1889), but he was unable to examine specimens of the third species, *Symmetropleura plana* (Walker, 1869). Now, the availability of the above listed specimens allows to propose the change of the taxonomic status of this species.

Characters of the genus *Symmetropleura* are: fastigium of vertex triangular and sulcate; pronotum disc flat, with lateral excisions; tegmina wide with rounded hind margin or narrow with straight hind margin; fore and mid femora with ventral inner spines, hind femora with double row of ventral spines. Fore and mid tibiae dorsally unarmed or with some spinules; cerci long, in-curved and pointed; male sub-genital plate short with rounded posterior margin or (in *Symmetropleura africana*) long with triangular apex; styli absent; ovipositor longer than pronotum, not much curved, sharp, with upper and lower apices serrate; female sub-genital plate triangular, just concave. In the description Brunner von Wattenwyl (1878) referred mainly to *Symmetropleura laevicauda*, both within the text and in the figure 73; thus, by subsequent designation, Kirby (1906) established *Symmetropleura laevicauda* as the type-species of the genus. Massa (2015) transferred *Symmetropleura africana* to the genus *Symmetrokarschia*, on the basis of its peculiar characters: pronotum disc with regular impressed punctures, fastigium of vertex compressed, narrower than first antennal segment, sulcate above, eyes oval, prominent; absence of fronto-genal carinae; pronotum just depressed, fore part with just definite lateral carinae, central and hind parts with vague lateral carinae; fore margin slightly concave, posterior margin rounded; surface dotted, matt; fore coxae with a long spine, fore tibiae with open tympanum on each side, furrowed on upper border; fore and mid femora with 3-5 spines, hind femora with 5-8 inner ventral and 6-7 outer ventral spines; fore and mid tibiae with 1 dorsal and 1 ventral spur, hind tibiae with 3 apical spurs on each side; male tenth tergite laminate and protruding, with straight posterior margin, cerci little in-curved, with flat apex and pointed, sub-genital plate long, narrow, with obtuse and short cut apex, styli absent. Ovipositor well developed, sharply bent upwards near the base, shorter than pronotum, with upper border and apex of lower border finely serrate, sub-genital plate triangular and pointed. Tegmina wide and oval, with rounded hind border more pronounced in female than in male.

**Figures 26–30.**
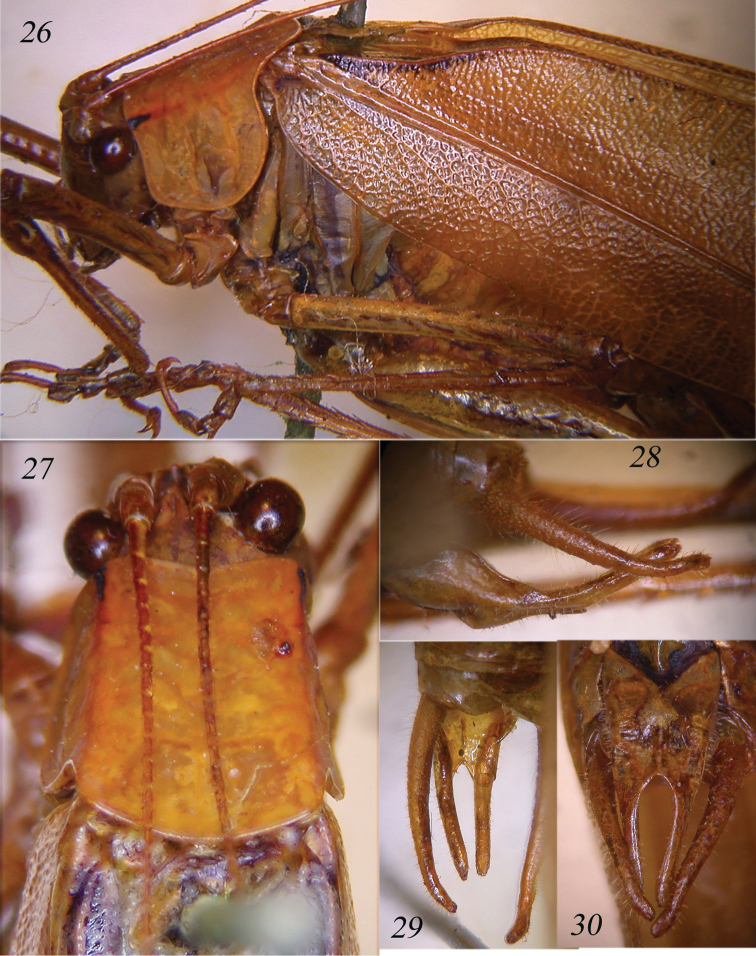
*Symmetrokarschia plana* comb. n. Lateral view of head, pronotum and tegmina of male (**26**); dorsal view of head and pronotum (**27**); lateral (**28**) and dorsal view (**29**) of cerci and appendices of male sub-genital plate; sub-genital plate of male and cerci (**30**).

Characters of *Symmetropleura plana* are testaceous-green, smooth, rather stout; head nearly as broad as the pronotum, with a short keel between eyes; front erect. Fastigium of vertex compressed, narrower than first antennal segment, sulcate above (Fig. 27). Eyes tawny, nearly round, rather prominent; absence of fronto-genal carinae. Disk of the pronotum flat, slightly widening hindward, with an abbreviated curved transverse line in middle; lateral keels just defined, each accompanied by an ochraceous line; fore margin slightly excavated; sides and hind margins slightly rounded, surface matt, characterized by a right and a left black spots on fore margin (Figs 26–27). Legs long, slender; fore coxae armed, fore tibiae with open tympanum on each side, furrowed on upper border. Fore tibiae with 6 inner + 1 spur and 7 outer spines + 1 spur on ventral margin, 3 outer spines + 1 spur on dorsal margin; mid tibiae with 8 inner + 1 spur and 10 outer spines + 1 spur on ventral margin, 2-3 inner and 7 outer spines + 1 spur on dorsal margin (on the whole both fore and mid tibiae have 1 dorsal and 2 ventral spurs); hind tibiae with 3 apical spurs on each side. Fore femora with 3-4 spines on each side of ventral margin, mid femora with 5 outer spines on ventral margin, unarmed on inner ventral margin. Hind femora with 3-4 small spines on each side of ventral margin. Fore wings rather narrow, with a ferrugineous streak along the anal vein and another nearer to base of hind margin (Fig. 26); interno-medial vein abruptly curved to the hind margin near tip; branch of externo-medial vein forked; veinlets very numerous, minute and irregular. Hind wings pellucid, longer than fore wings, green and with texture as in fore wings along apical part of costa; veins white. Male tenth tergite laminate with straight posterior margin (Fig. 29), cerci and lower appendages rounded at tips, nearly cylindrical, the former more curved than the latter, cerci very long, up-curved with flat and just pointed apex (Figs 28–29); sub-genital plate very long, narrow, with two very long appendices, just shorter than cerci; styli absent (Fig. 30).

####### Diagnosis.

Differences from *Symmetrokarschia africana* are the absence of evident lateral carinae on metazona of pronotum, narrow tegmina, and fore and mid tibiae with 3 spurs. Considering the high variability of some characters found in other genera of African Phaneropterinae, it seems reasonable to consider also *Symmetrokarschia plana* as belonging to the genus *Symmetrokarschia*, and to exclude the genus *Symmetropleura* definitively from the African fauna.

###### Preussia lobatipes

Taxon classificationAnimaliaOrthopteraTettigoniidae

Karsch, 1890

####### Material examined.

Cameroon, Barombi Station (holotype ♀) (MfN); Ivory Coast, Man, Mt. Tonkoui (1200 m) 28.VI–1.VII.2014 (UV trap), P. Moretto (♂) (BMCP).

####### Remarks.

*Preussia lobatipes* was described from one female from Barombi Station (Cameroon) and considered by the author as being related to *Symmetropleura africana* (see above); the male was described one year later from the same locality (Karsch 1891). Griffini (1908) recorded it from Mukonje Farm (Cameroon), Leroy (1985) from Central African Republic and Naskrecki (2008, 2009) from Ghana. This from Ivory Coast is new and the westernmost record known till now, Mt. Tonkoui is at NW of Man, next to the border with Guinea.

##### Genera *Dapanera* Karsch, 1889

Karsch, 1889. Berlin Ent. Z. 32: 423, 441

###### Gonatoxia

Taxon classificationAnimaliaOrthopteraTettigoniidae

Karsch, 1889 syn. n.

Gonatoxia Karsch, 1889. Berlin Ent. Z. 32: 423, 441

####### Remarks.

**Figures 31–38.**
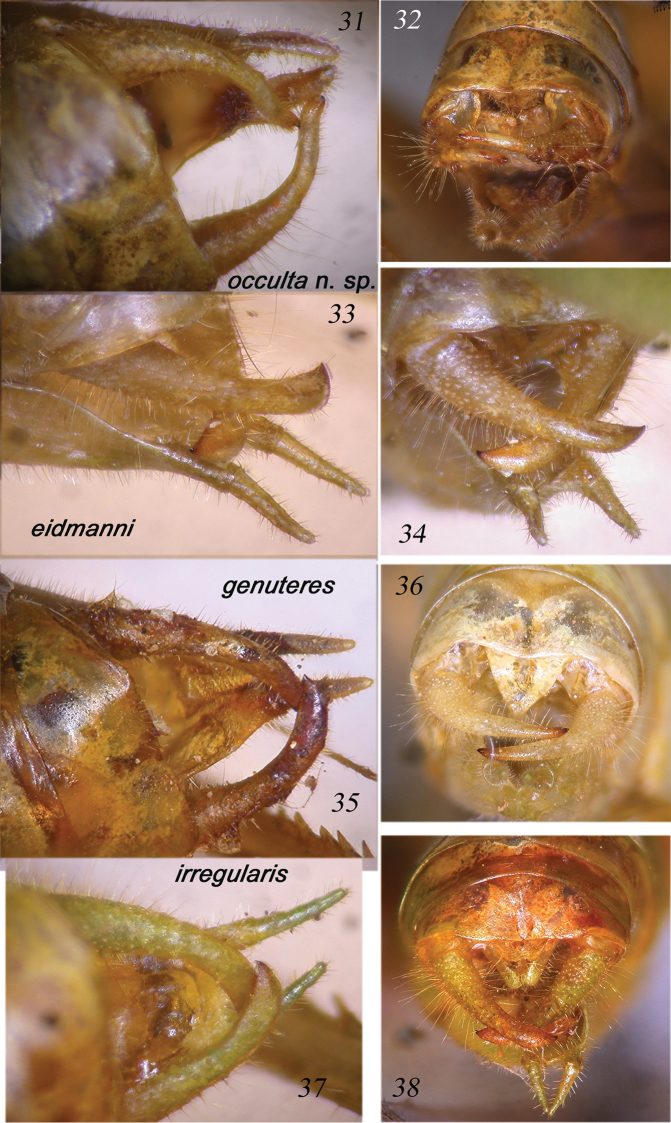
Lateral and dorsal view of male cerci and sub-genital plate of *Dapanera occulta* sp. n. (**31–32**), *Dapanera eidmanni* (**33–34**), *Dapanera genuteres* (**35–36**) and *Dapanera irregularis* (37–38).

**Figures 39–42.**
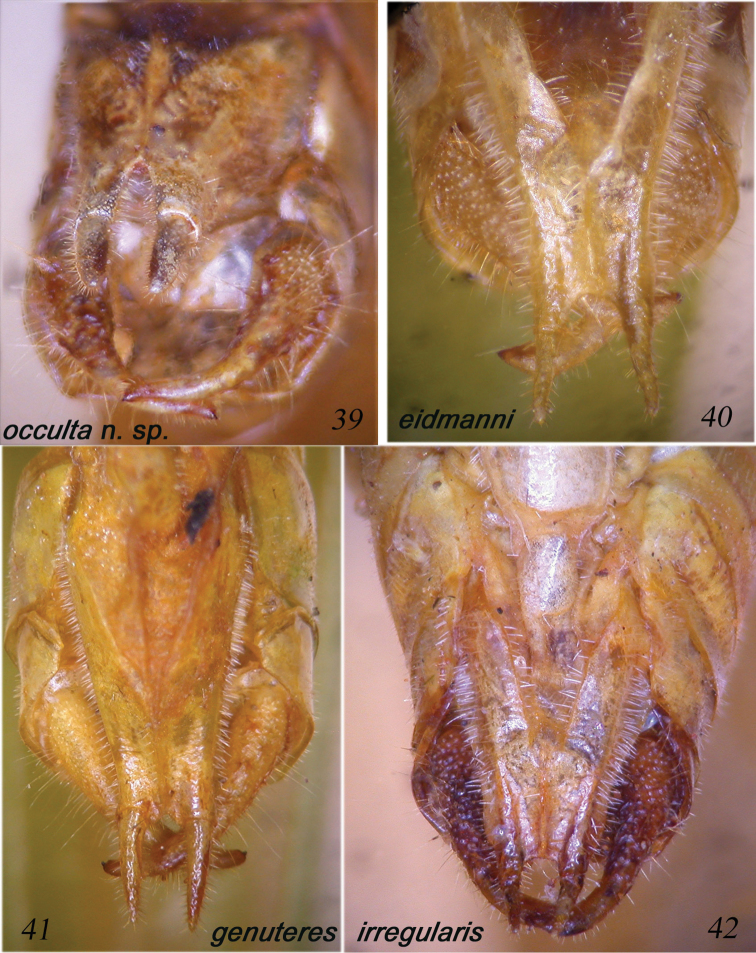
Male sub-genital plate of *Dapanera occulta* sp. n. (**39**), *Dapanera eidmanni* (**40**), *Dapanera genuteres* (**41**) and *Dapanera irregularis* (**42**).

**Figures 43–46.**
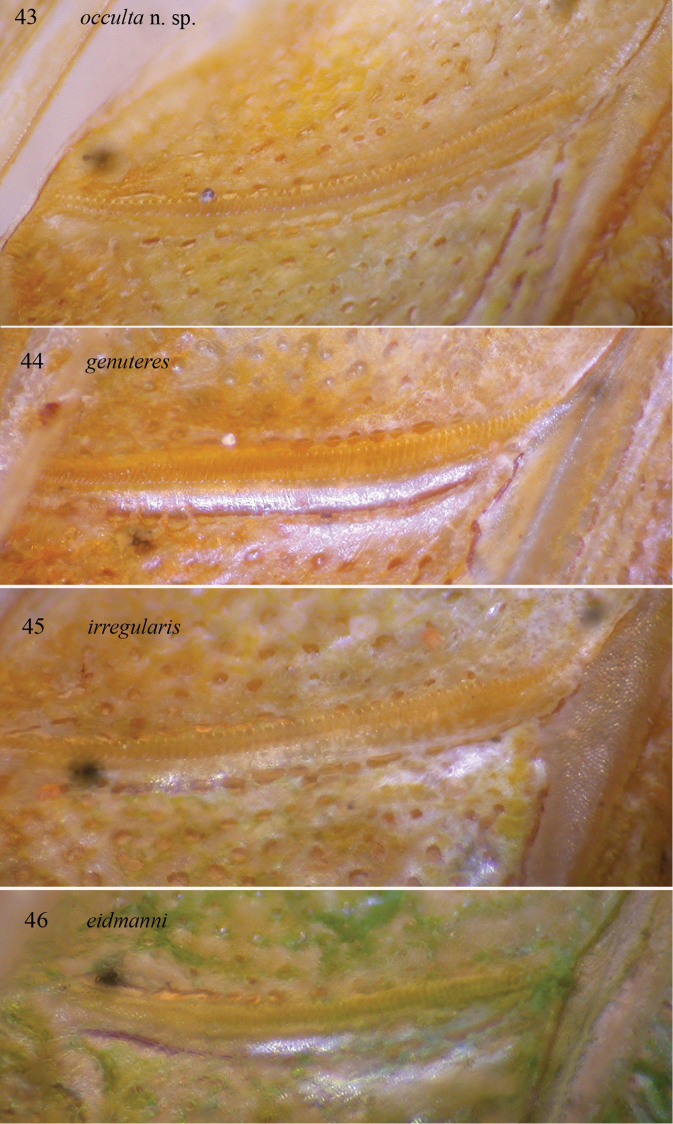
Stridulatory file of left tegmen of male of *Dapanera occulta* sp. n. (**43**), *Dapanera genuteres* (**44**), *Dapanera irregularis* (**45**) and *Dapanera eidmanni* (**46**).

**Figures 47–49.**
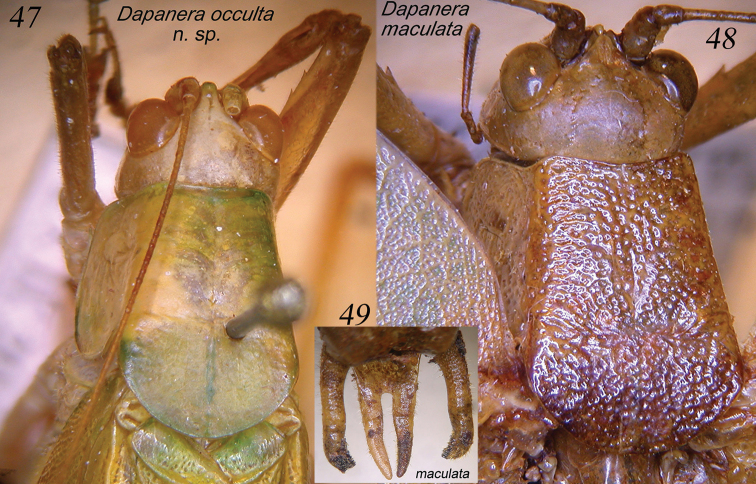
Dorsal view of head and pronotum of males of *Dapanera occulta* sp. n. (**47**) and *Dapanera maculata* comb. n. (**48**); cerci and sub-genital plate of male of *Dapanera maculata* comb. n. (**49**).

The genus *Dapanera* was erected by Karsch (1889) and is characterized by stout and long styli. Karsch (1889) described *Dapanera genuteres*, and later (Karsch 1890) *Dapanera irregularis*, very similar to the previous species, but with different cerci shape and length of styli (shorter than in *Dapanera genuteres*). Further, Griffini (1908), Sjostedt (1912) and Massa (2013) have highlighted that *Dapanera irregularis* is smaller than *Dapanera genuteres* and has shorter ovipositor. The genus *Dapanera* contains another species, *Dapanera eidmanni* (see below); all the species are morphologically very similar, with the exception of the shape of the male sub-genital plate, cerci, styli length and stridulatory files (Figs 31–49). The genus *Gonatoxia* was described by Karsch (1889) on the same page as *Dapanera* with the following differences: fastigium of vertex not sulcate, tegmina wider and genicular lobes of hind femora with a spine. However, the fastigium of *Gonatoxia* may be sulcate as shown in Fig. 48 and also the genicular lobes of the hind femora of *Dapanera* may present a small spine. Remaining as difference the width of tegmina, a variable character within the same genus in Phaneropterinae, it seems rather evident that *Dapanera* and *Gonatoxia* are synonyms, with priority for *Dapanera*, described by Karsch (1889) before *Gonatoxia*. Thus, the two *Gonatoxia* species become *Dapanera maculata* (Karsch, 1889), comb. n. and *Dapanera immaculata* (Karsch, 1889), comb. n. Species described in the genus *Gonatoxia* are known from East Africa, while those of the genus *Dapanera* from West and central Africa. A further new species of *Dapanera* is here described from the Central African Republic.

###### Dapanera maculata

Taxon classificationAnimaliaOrthopteraTettigoniidae

(Karsch, 1889) comb. n.

Figs 48 , 49

####### Material examined.

Somalia (1♀) (MNCN); Somalia, Mogadishu (1♂) (MZR).

####### Remarks.

*Dapanera maculata* has very stout styli, stout cerci (Fig. 49) and punctured pronotum (Fig. 48). It is distributed in Somalia, Kenya and Tanzania (Hemp 2013).

###### Dapanera eidmanni

Taxon classificationAnimaliaOrthopteraTettigoniidae

Ebner, 1943

Figs 33 , 34 , 40 , 46

####### Material examined.

Ivory Coast, Man, Mt. Tonkoui (1200 m) 1-4.VII.2014 (UV trap), P. Moretto (1♂) (BMCP).

####### Remarks.

Described from Bioko, Fernando Poo (Equatorial Guinea) (Ebner 1943), it is distinguished from *Dapanera irregularis* Karsch, 1890 mainly by the cercus shape (Figs 33, 34, 40). This is the first record of the species since its description. The record from Ivory Coast extends its distribution remarkably eastwards. *Dapanera eidmanni* is also distinguished from *Dapanera irregularis* by its sub-genital plate, which is similar to that of *Dapanera genuteres*, little divided and with slender and long styli (compare Fig. 40 with 41 and 42). However, the cerci of *Dapanera eidmanni* are stout, more similar to those of *Dapanera irregularis* (compare Figs 33–34 with 35–38).

###### Dapanera occulta sp. n.

Taxon classificationAnimaliaOrthopteraTettigoniidae

http://zoobank.org/C1384DAE-60D9-441E-A0DE-051D695CD4BF

Figs 31 , 32 , 39 , 43 , 47

####### Material examined and depository.

Central African Republic, Dzanga-Ndoki National Park, Ndoki, Lake 1, UV trap 1, 02°28'40.5N, 016°13'02.6E, 31.I.-2.II.2012, P. Moretto (♂ holotype) (MSNG); same data (♂ paratype); Central African Republic, Dzanga-Ndoki National Park, Ndoki, Lake 1, UV trap 1, 02°28'40.5N, 016°13'02.6E, 10–11.II.2012, P. Moretto (♂ paratype); same data, 11–12.II.2012 (2♂ paratypes); same data, 20–23.II.2012 (♂ paratype); Central African Republic, Dzanga-Ndoki National Park, Ndoki, border of Lake 1, UV trap 02°28'51.0N, 016°13'04.5E, 13–14.II.2012, P. Moretto (♂ paratype) (BMCP).

####### Colour.

Yellow-green. Femora yellow with or without longitudinal brown stripe on outer side.

####### Description.

Male. Medium sized. Head and antennae: fastigium of vertex narrow, furrowed above, separated from fastigium of frons that is tuberculated. Eyes rounded, well projecting (Fig. 47). Legs comparatively long. Fore coxae armed with a well-developed spine. Fore tibiae furrowed on upper margin, distinctly widening above tympanum, which is closed on inner and open on outer side. Fore femora armed on inner ventral margin with 3-4 spines, fore tibiae with 3 spines plus 1 spur on inner and outer ventral margins, 1 spur on outer dorsal margin, mid femora armed with 5 spines on outer ventral margin, mid tibiae with 8 on outer and 4-5 spines on inner ventral margins, plus 1 spur on each side, hind femora armed with 7-8 spines on outer and inner ventral margins, hind tibiae with many spines on ventral and dorsal margins and 3 spurs on each side. Thorax: pronotum little narrowing anteriorly, flat above, anterior margin straight, posterior margin rounded, humeral sinus evident, lobes of pronotum rounded. Tegmina comparatively wide with rounded apices. Wings longer than tegmina. Stridulatory region of left tegmen narrow, stridulatory file curved and composed of 70–75 teeth (Fig. 43). Abdomen: tenth tergite with straight hind margin; sub-genital plate long and deeply divided into two lobes; styli stout and long (Figs 31–32). Cerci long, thin, in-curved and sinuous, with small apical spine, longer than sub-genital plate (Figs 31, 32, 39).

####### Measurements.

Males. Body length: 21.6–24.1; pronotum length: 5.5–5.6; pronotum height: 4.6–4.8; hind femur: 18.7–20.8; tegmina: 32.9–34.9.

####### Diagnosis.

*Dapanera occulta* sp. n. is mainly characterized by its cerci that in the other species of the genus *Dapanera* are stout and never sinuous; in addition, the styli are stout and short, while in *Dapanera genuteres* and *Dapanera eidmanni* they are slender and longer (Figs 33–36); the sub-genital plate is deeply divided, more than in *Dapanera irregularis*, *Dapanera eidmanni* and *Dapanera genuteres* (in the latter two species it is very little divided) (Figs 39–42). The stridulatory files of *Dapanera genuteres*, *Dapanera eidmanni* and *Dapanera irregularis* are less curved, also composed of 70–75 teeth or more; the teeth are larger in *Dapanera genuteres* and *Dapanera irregularis* than in *Dapanera occulta* sp. n., while in *Dapanera eidmanni* as small as in *Dapanera occulta* sp. n. (Figs 43–46).

Female. Unknown.

####### Etymology.

From Latin (*occulta* = hidden), female adjective; the series of specimens remained unidentified and hidden in a box containing long series of *Dapanera irregularis* and *Dapanera genuteres* collected in the same localities during the same expedition (see Massa 2013).

###### Cestromoecha laeglae sp. n.

Taxon classificationAnimaliaOrthopteraTettigoniidae

http://zoobank.org/589D3B04-164C-4011-B240-14A9DD25F1C1

Figs 50–54 , 55–57

####### Material examined and depository.

Ivory Coast, Tuba, Biémasso (441 m), 8°04'00.09"N, 7°32'59.96"W (UV trap) 9.VII.2014, P. Moretto (♂ holotype) (MSNG); same locality, 7–11.VII.2014, P. Moretto (♂ paratype, ♀ allotype); same locality, 9.VII.2014, P. Moretto (♂ paratype) (BMCP).

The genus *Cestromoecha* Karsch, 1893 is related to *Poreuomena* Brunner von Wattenwyl, 1878, which also lives in central-western Africa and differs from it chiefly in the shape of the male tenth tergite, being slightly bilobate or rounded, in the male sub-genital plate, being deeply bilobate, and in the shape of the cerci. Styli are absent. Five species are known, *Cestromoecha crassipes* (Karsch, 1890), *Cestromoecha tenuipes* (Karsch, 1890), *Cestromoecha mundamensis* Karsch, 1896, *Cestromoecha longicerca* Massa, 2013 and *Cestromoecha magnicerca* Massa, 2013. Here a sixth species is described.

####### Colour.

Brown or green, stridulatory area of left tegmen and area below it black. Small black spots are present on posterior margins of tegmina. Two longitudinal parallel dark lines are present on outer surface of hind femora.

####### Description.

**Figures 50–54.**
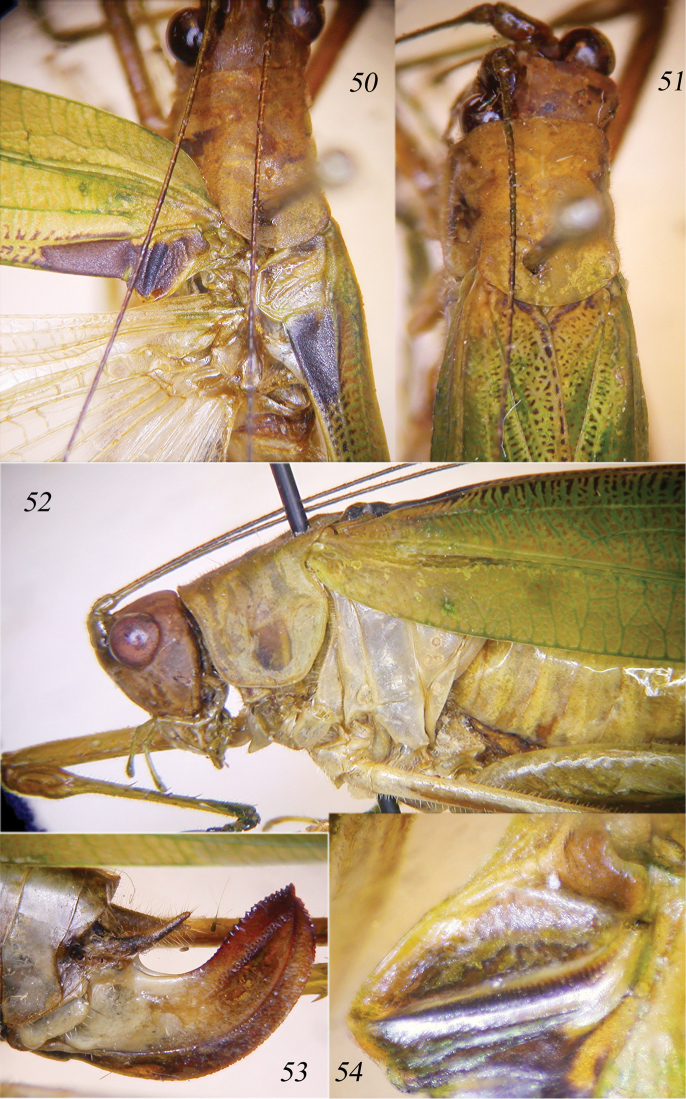
*Cestromoecha laeglae* sp. n. Dorsal view of head, pronotum and tegmina of male (**50**) and female (**51**); lateral view of head, pronotum and tegmina of male (**52**); lateral view of ovipositor (**53**); stridulatory file of left tegmen of male (**54**).

**Figures 55–57.**
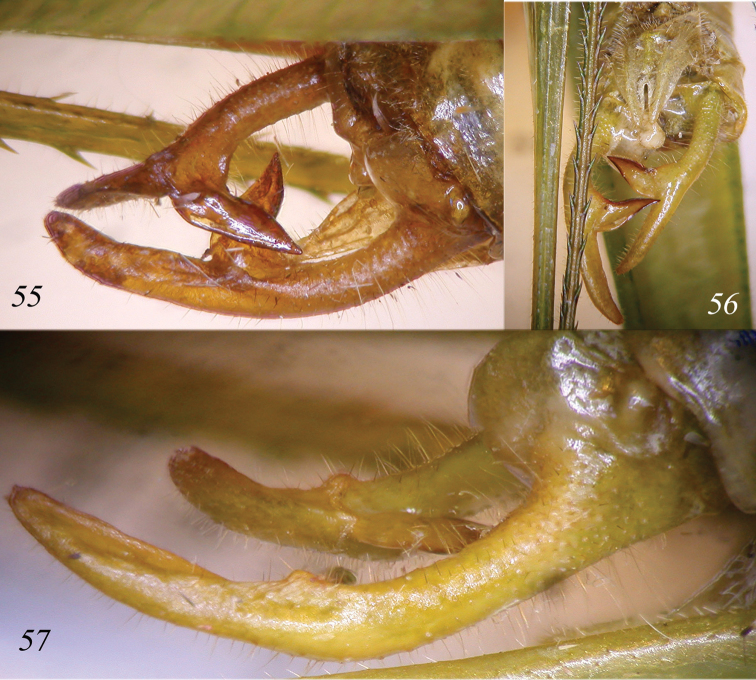
*Cestromoecha laeglae* sp. n. Dorsal view of male cerci (**55**); sub-genital plate and cerci of male (**56**); lateral view of cerci (**57**).

Male. Diagnostic characters of the genus. Eyes round (Fig. 52), fastigium of vertex triangular, sulcate. Fore coxae armed, fore and mid femora with 4–5 very small spines^2^, fore tibiae with 3 ventral spines + 1 spur on each side, mid tibiae with 6–7 ventral spines + 1 spur on each side, hind tibiae with 3 spurs on each side. Ventral margins of hind femora with 2 small basal spines. Tegmina narrow, stridulatory area of left tegmen black and straight (Fig. 50); stridulatory file down-curved with ca. 50 teeth, distal part with asymmetrical and widely spaced teeth (Fig. 54). Tenth tergite slightly bilobate. Cerci stout, long and in-curved, with basal part rounded and apical part flattened and pointed; in middle with a well-developed flattened large inner spine, blackish at tip. Sub-genital plate concave, triangular and long, with a deep concavity, processes rather parallel (Figs 55–57).

Female. As male, but without blackish markings and with only brown spots (Fig. 51). Ovipositor up-curved and provided with small denticles on the upper and lower margins (Fig. 53).

####### Measurements.

Males. Body length: 18.5–19.4; pronotum length: 4.0–4.2; pronotum height: 3.4–3.6; hind femur: 18.2–20.7; tegmina: 26.4–27.5. Female. Body length: 21.7; pronotum length: 4.0; pronotum height: 3.4; hind femur: 20.8; tegmina: 29.4; ovipositor: 6.1.

####### Diagnosis.

*Cestromoecha laeglae* sp. n. is related to *Cestromoecha magnicerca*. The cerci of the male are stout, long and in-curved, with the basal part rounded and the apical part flattened and pointed; a wide flattened inner spine arises from its middle; in *Cestromoecha magnicerca* the cerci have trifid apices. The sub-genital plate is concave, but not long, with parallel processes, very similar to those of *Cestromoecha magnicerca*. The stridulatory file of *Cestromoecha laeglae* sp. n. is also similar to that of *Cestromoecha magnicerca* with distal part with less and more widely spaced teeth than the proximal part (see Massa 2013).

####### Etymology.

Laegla is the nickname of Giovanna Varrica, to whom this species is dedicated.

## Supplementary Material

XML Treatment for Ruspolia differens

XML Treatment for Thyridorhoptrum senegalense

XML Treatment for Thyridorhoptrum carbonarium

XML Treatment for Horatosphaga leggei

XML Treatment for Horatosphaga linearis

XML Treatment for Horatosphaga aethiopica

XML Treatment for Symmetrokarschia plana

XML Treatment for Preussia lobatipes

XML Treatment for Gonatoxia

XML Treatment for Dapanera maculata

XML Treatment for Dapanera eidmanni

XML Treatment for Dapanera occulta

XML Treatment for Cestromoecha laeglae

## Footnotes

1 Recently Heller et al. (2014) proposed to consider Phaneropterinae at the family level, but Braun (2015) and Song et al. (2015) have shown that they have to be considered a sub-family of Tettigoniidae.

2 Differing from what has been reported by Massa (2013), species of genera *Cestromoecha* and *Poreuomena* have 4–5 very small spines on ventral margins of fore and mid femora.
